# A study on the role of Taxifolin in inducing apoptosis of pancreatic cancer cells: screening results using weighted gene co-expression network analysis

**DOI:** 10.18632/aging.205500

**Published:** 2024-02-01

**Authors:** Shao-Jie Chen, Li-Kun Ren, Xiao-Bin Fei, Peng Liu, Xing Wang, Chang-Hao Zhu, Yao-Zhen Pan

**Affiliations:** 1 Department of Hepatobiliary Surgery, Affiliated Hospital of Guizhou Medical University, Guiyang, China; 2 School of Clinical Medicine, Guizhou Medical University, Guiyang, China; 3 Department of Hepatobiliary Surgery, Affiliated Cancer Hospital of Guizhou Medical University, Guiyang, China

**Keywords:** PAAD, weighted gene co-expression network analysis (WGCNA), Taxifolin

## Abstract

Pancreatic adenocarcinoma (PAAD) is a frequent malignant tumor in the pancreas. The incomplete understanding of cancer etiology and pathogenesis, as well as the limitations in early detection and diagnostic methods, have created an urgent need for the discovery of new therapeutic targets and drugs to control this disease. As a result, the current therapeutic options are limited. In this study, the weighted gene co-expression network analysis (WGCNA) method was employed to identify key genes associated with the progression and prognosis of pancreatic adenocarcinoma (PAAD) patients in the Gene Expression Profiling Interactive Analysis (GEPIA) database. To identify small molecule drugs with potential in the treatment of pancreatic adenocarcinoma (PAAD), we compared key genes to the reference dataset in the CMAP database. First, we analyzed the antitumor properties of small molecule drugs using cell counting kit-8 (CCK-8), AO/EB and Transwell assays. Subsequently, we integrated network pharmacology with molecular docking to explore the potential mechanisms of the identified molecules' anti-tumor effects. Our findings indicated that the progression and prognosis of PAAD patients in pancreatic cancer were associated with 11 genes, namely, DKK1, S100A2, CDA, KRT6A, ITGA3, GPR87, IL20RB, ZBED2, PMEPA1, CST6, and MUC16. These genes were filtered based on their therapeutic potential through comparing them with the reference dataset in the CMAP database. Taxifolin, a natural small molecule drug with the potential for treating PAAD, was screened by comparing it with the reference dataset in the CMAP database. Cell-based experiments have validated the potential of Taxifolin to facilitate apoptosis in pancreatic cancer cells while restraining their invasion and metastasis. This outcome is believed to be achieved via the HIF-1 signaling pathway. In conclusion, this study provided a theoretical basis for screening genes related to the progression of pancreatic cancer and discovered potentially active small molecule drugs. The experimental results confirm that Taxifolin has the ability to promote apoptosis in pancreatic cancer cells.

## INTRODUCTION

Pancreatic cancer is a prevalent type of cancer, ranking seventh in global cancer mortality [1–3]. Incidence of pancreatic cancer is expected to rise with population growth, accelerated ageing and the spread of Westernized lifestyles. Pancreatic cancer has a poor prognosis, mainly due to delayed diagnosis and missed surgery as well as a lack of specific biomarkers and targeted drugs [4, 5]. Identifying novel prognostic biomarkers and formulating improved therapeutic approaches is therefore essential.

Natural products play an important role in developing anti-cancer drugs, primarily because they’re readily available and possess potent bioactivity, but the high cost and long lead time restrict the development of new drugs [6–8]. With the development of gene chips and sequencing technology, we now have the potential to use bioinformatics and big data integration to identify key genes related to tumor progression and prognosis [9]. WGCNA is a systems biology method for characterizing gene association patterns in diverse samples, which are essential for understanding underlying biological mechanisms. It aims to identify highly synergistic gene sets and determine potential biomarker genes or therapeutic targets by considering their interconnectedness and association with the phenotype [10, 11]. Similarly, researchers have developed a gene expression database called the Connectivity Map (CMAP). which is based on the L1000 sequencing method that identifies highly co-altered genes and contains gene expression profiling data for many different drug treatments [12, 13]. Screening key tumor genes using WGCNA and then combining them with CMAP to identify effective drugs for treating tumors is an efficient approach to discover disease biomarkers and potential drugs with lower costs [14].

This study utilized WGCNA to filter key genes significantly associated with pancreatic cancer development and progression. The CMAP database was used in identifying relevant small molecule target drugs and selecting natural small molecule Taxifolin for further study. The experimental results provide evidence supporting Taxifolin’s potential to hinder pancreatic cancer growth.

## RESULTS

### Screening differential genes in gene expression profile data

**Figure 1:**
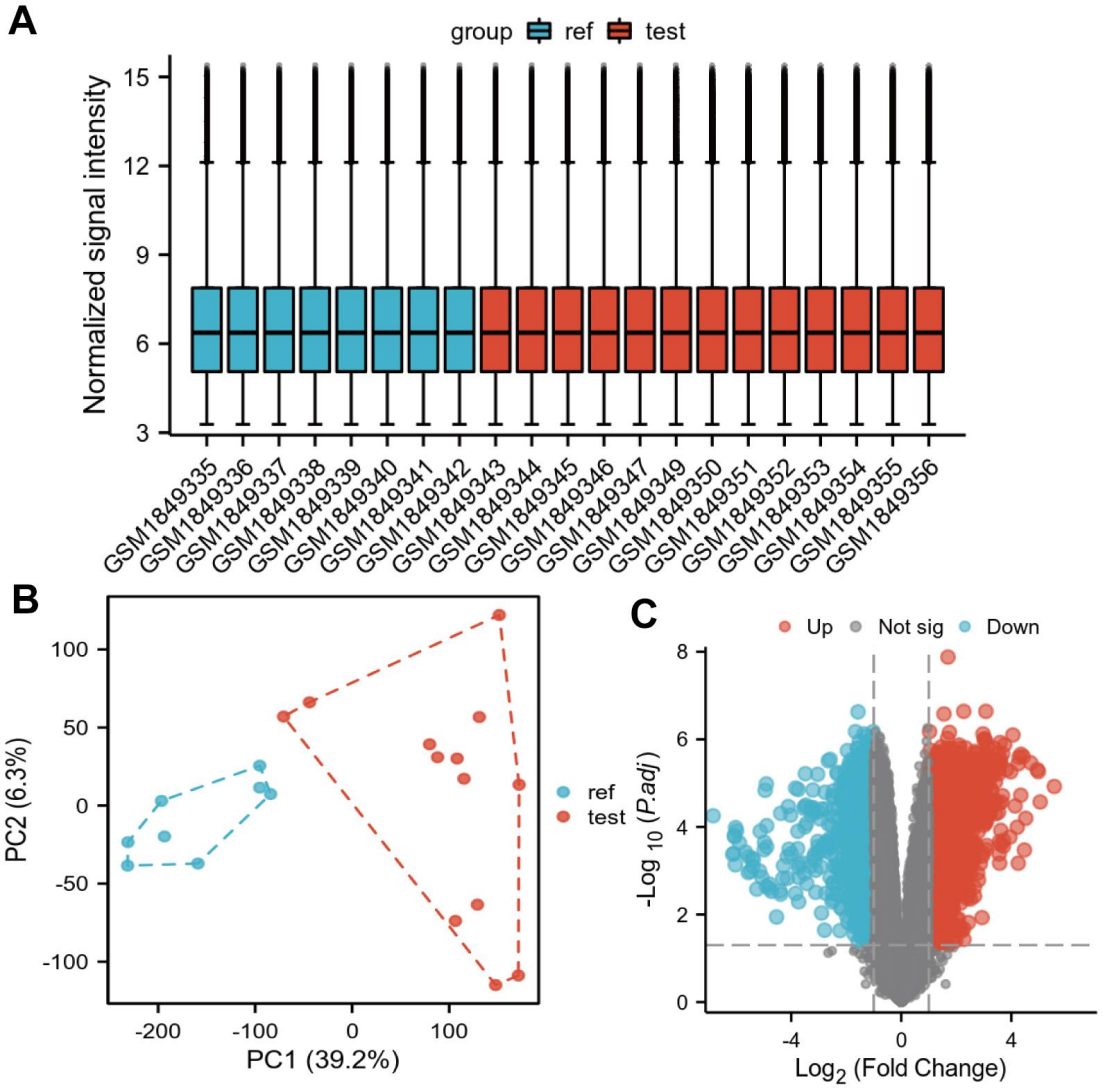
**Differentially expressed genes identified in the GSE71989 dataset.** (**A**) Box plot showing the correction across samples, (**B**) PCA plot showing the differences across samples after downscaling the high latitude data, and (**C**) volcano plot showing the genes that showed differential expression in GSE71989. Up-regulated genes are indicated by red nodes and down-regulated genes are indicated by green nodes.

In Figure 1A, the box plots showed that the correction was good and there were no outliers among the samples, the PCA plots in Figure 1B showed that the differential genes were expressed significantly differently in tumor samples and normal samples, and the volcano plots showed the differentially expressed genes in the GSE71989 dataset (Figure 1C). After eliminating duplicated genes, a total of 3292 up-regulated genes and 748 down-regulated genes were found. The top five up-regulated genes were INHBA, S100P, CTHRC1, SULF1 and COL8A1, and the top five down-regulated genes were ALB, SYCN, GCG, CTRL and PNLIRP1.

### Analysis of gene ontology (GO) enrichment and the KEGG signaling pathway was conducted for the differentially expressed genes

**Figure 2:**
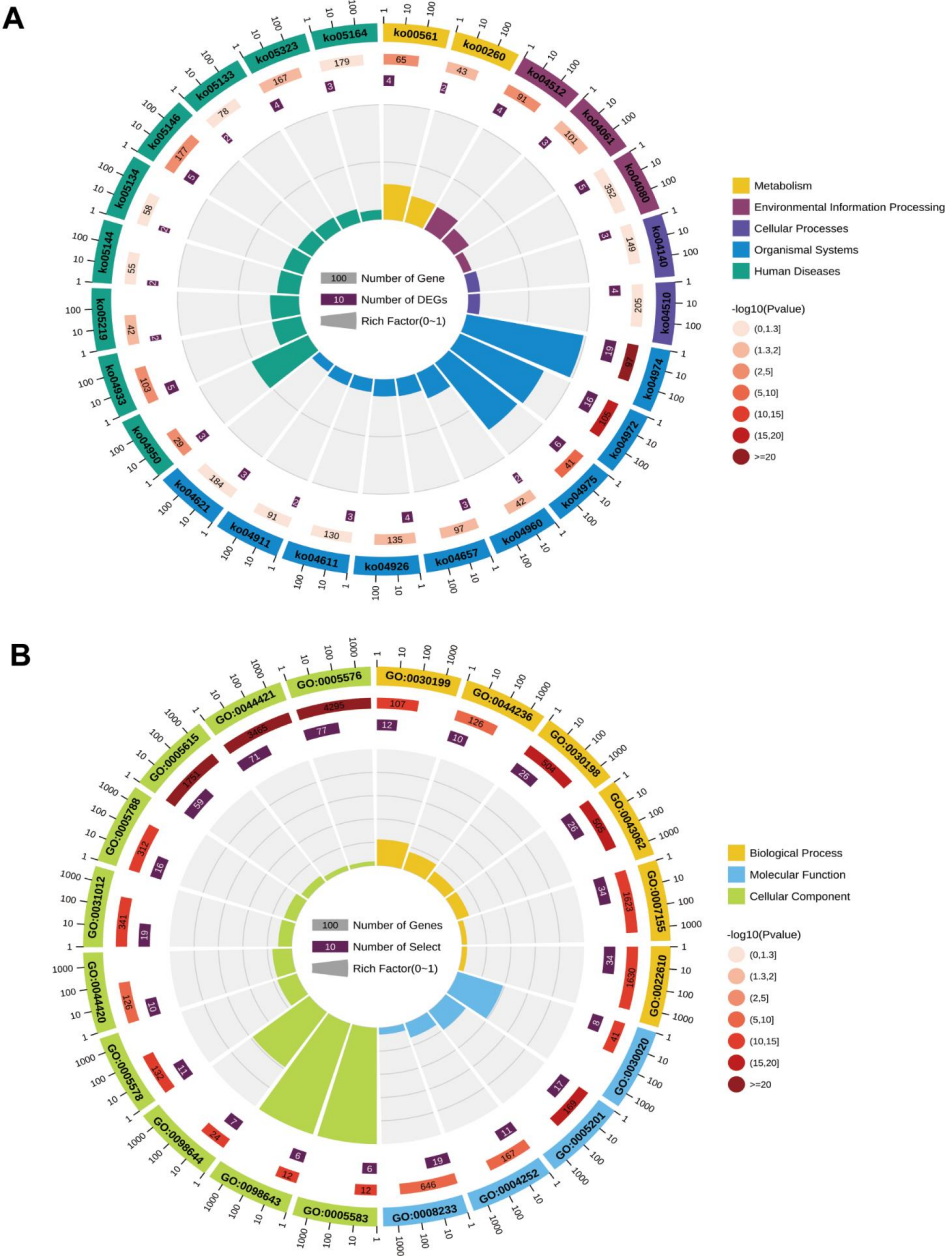
Circle plots of KEGG pathway analysis (**A**) and GO enrichment analysis (**B**) for differentially expressed genes in the GSE71989 dataset.

We performed pathway and Gene Ontology (GO) enrichment analysis utilizing the first 100 differential gene set previously obtained. The KEGG pathway analysis results showed that protein digestion and absorption, pancreatic fluid secretion, fat digestion and absorption, AGE-RAGE signaling pathway in diabetic complications, and mature diabetes mellitus were significantly enriched (Figure 2A). The GO enrichment analysis displayed significant enrichment in biological processes like extracellular matrix organization, extracellular structural organization, collagen fiber organization, and cell adhesion. Additionally, the cellular components that were enriched included extracellular space, extracellular region, extracellular regional part, and molecular functions like the structural components of the extracellular matrix. The top 20 enrichment results were selected based on the P-value and displayed in circle diagrams (Figure 2B).

### Weighted co-expression network construction and key module identification

**Figure 3:**
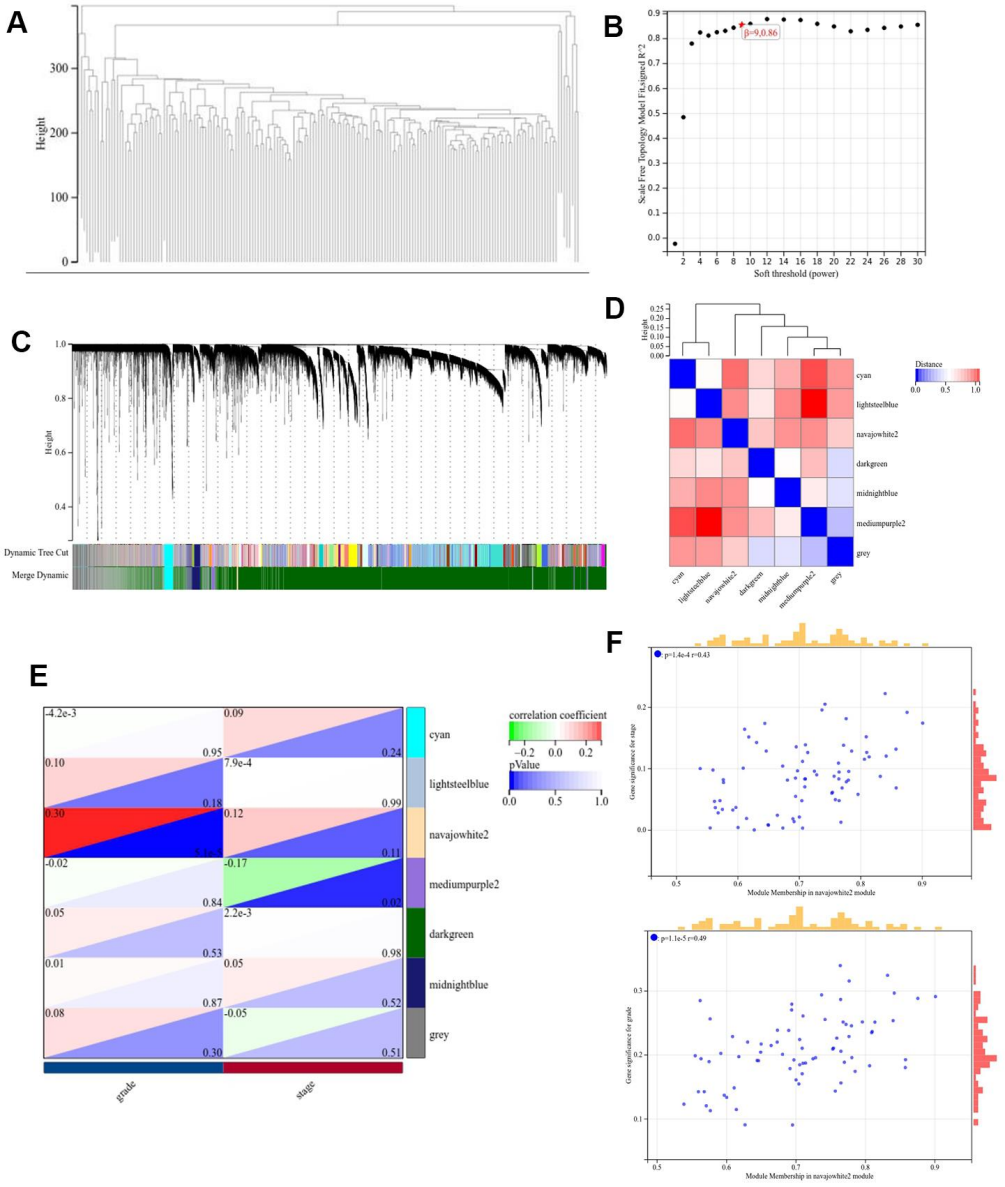
**Construction of the WGCNA co-expression module.** (**A**) Clustering dendrogram of the module feature genes. (**B**) Scale-free fit index analysis. (**C**) Clustering dendrogram of differentially expressed genes. (**D**) Network heatmap of modules. (**E**) Heat map demonstrating the correlation between the genes associated with module features and the staging and grading of pathology. (**F**) Scatterplot of module signature genes in the Navajowhite module.

The pancreatic cancer dataset from TCGA was downloaded from the UCSC database to create the input dataset used in WGCNA. The clustering dendrogram in the sample (Figure 3A) indicates an absence of significant differences in WGCNA between the samples. On the basis of the scale-free topological model and the average connection for the WGCNA analysis, the optimal soft threshold was set to 9 (Figure 3). The minimum size of the gene dendrogram was fixed at 30 in order to group genes with near-identical expression profiles into gene modules using the TOM-based dissimilarity metric and average linkage Hierarchical Clustering. The sensitivity was fixed to three, in addition, 7 co-expression modules were obtained by merging modules with a distance of smaller than 0.5. Noticeably, the gray modules were considered as a collection of genes that could not be assigned to any other module, and genes in gray modules were removed from subsequent analyses (Figure 3C). The interactions of the seven modules were analyzed using a network heat map. The results indicate a high degree of independence among modules, as evidenced by the fact that they were found to be independent of one another (Figure 3D). Moreover, the Navajowhite module’s ME exhibited a significant correlation with the grading and staging of pancreatic cancer as compared to other modules (Figure 3E, 3F). Thus, we chose to examine 64 genes in the Navajowhite module more closely.

### Functional annotation and KEGG pathway enrichment of the Navajowhite module

**Figure 4:**
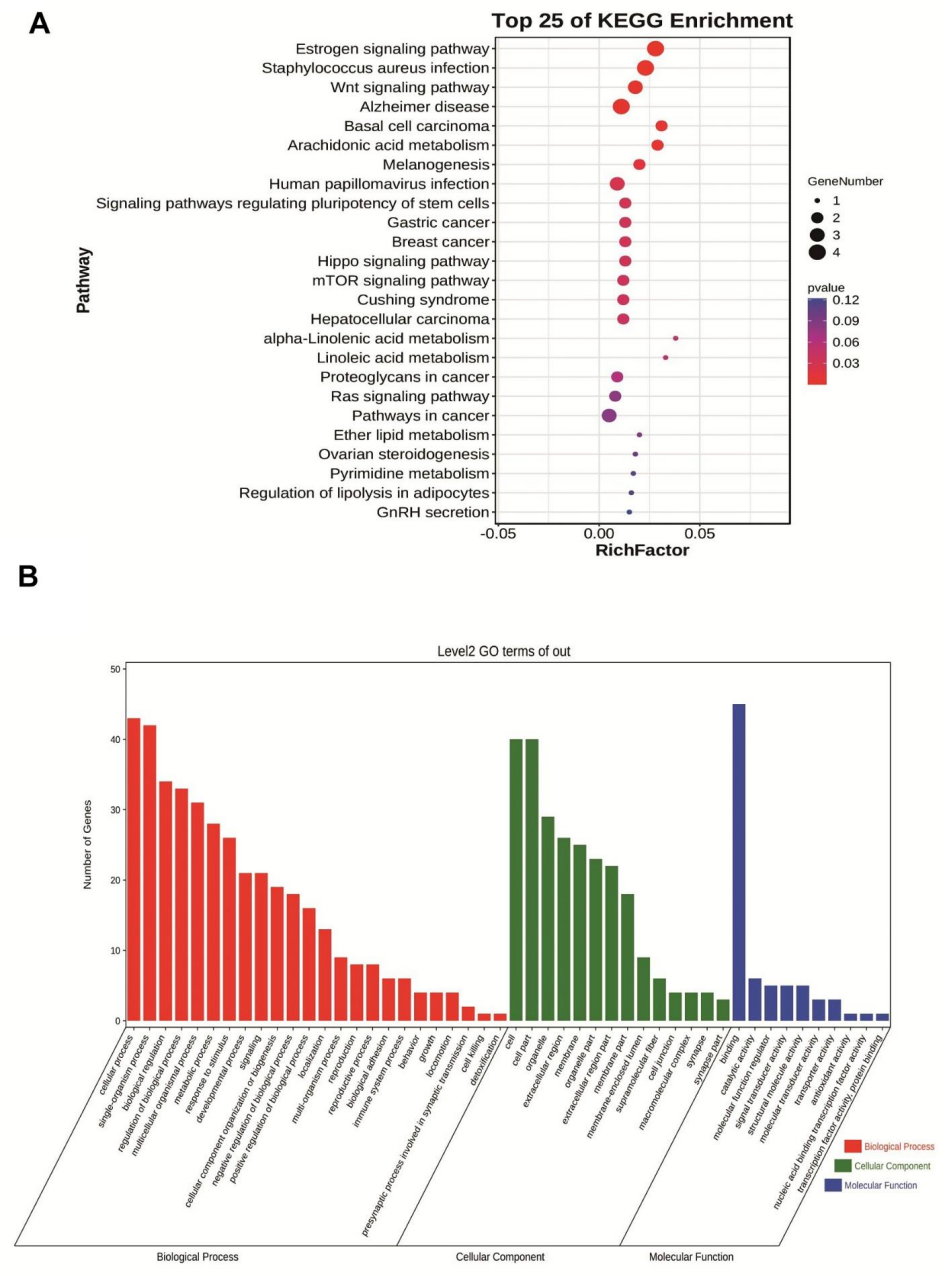
(**A**) Bubble plot of KEGG enrichment analysis of module feature genes in Navajowhite module. (**B**) Category histogram of the GO enrichment analysis of feature genes within the Navajowhite module.

We performed GO analysis and KEGG pathway enrichment on the above Navajowhite modules to investigate potential biological processes associated with pancreatic cancer. Figure 4A shows the KEGG pathway enrichment results, revealing significant pathways that include the estrogen signaling pathway, Staphylococcus aureus infection, the Wnt signaling pathway, Alzheimer’s disease and basal cell carcinoma. GO analysis of biological processes revealed that Navajowhite module genes are predominantly associated with epidermis development, keratinization, skin formation, and epithelium development (Figure 4B).

### Detection and validation of key genes for pancreatic cancer

**Figure 5:**
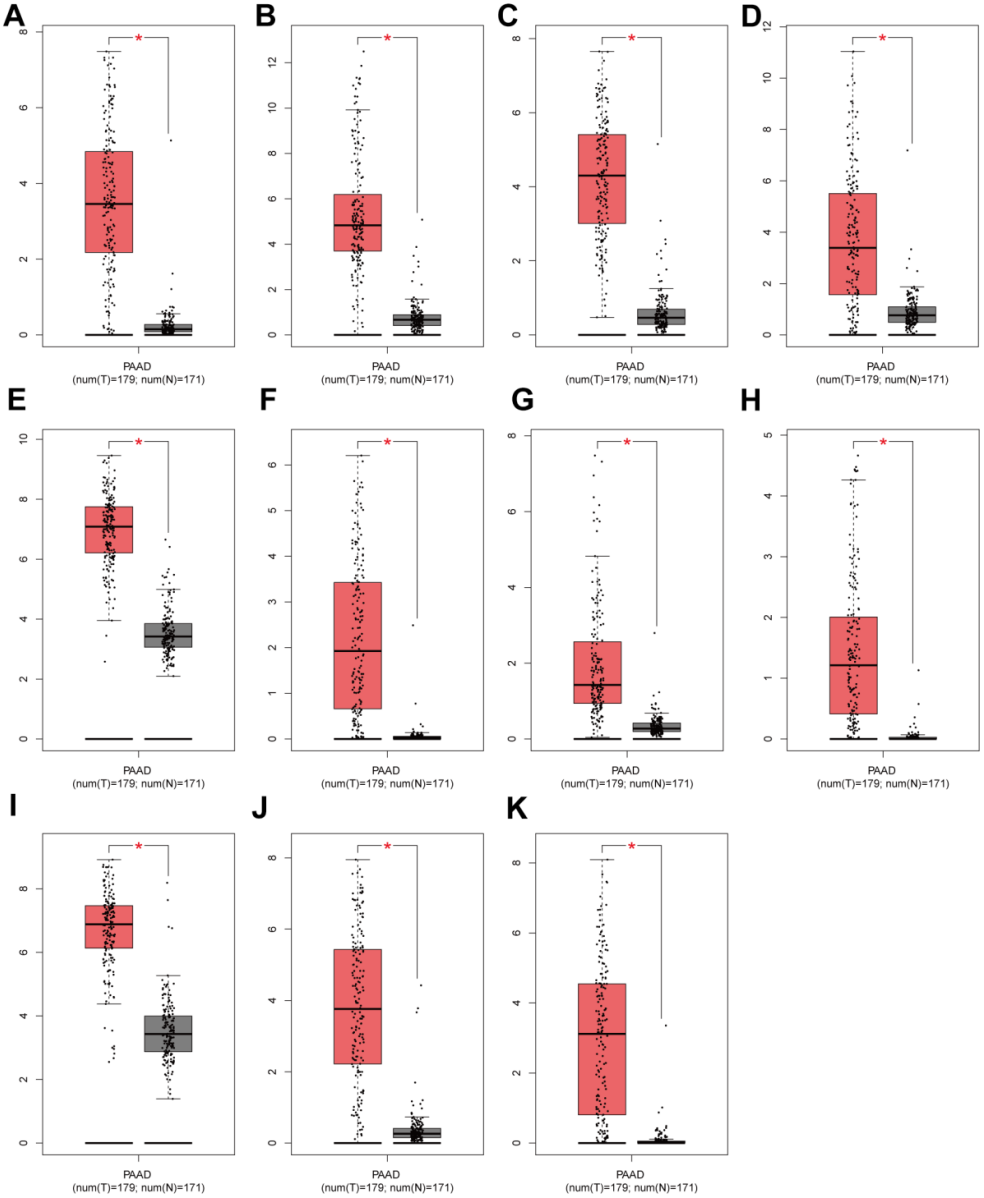
Validation of expression levels of individual core genes in normal pancreas and PAAD tissues from the GEPIA database (**A**–**K**) DKK1, S100A2, CDA, KRT6A, ITGA3, GPR87, IL20RB, ZBED2, PMEPA1, CST6 and MUC16 were significantly up-regulated in PAAD when compared to normal tissues (P < 0. 01). Red * indicates P < 0. 01.

**Figure 6:**
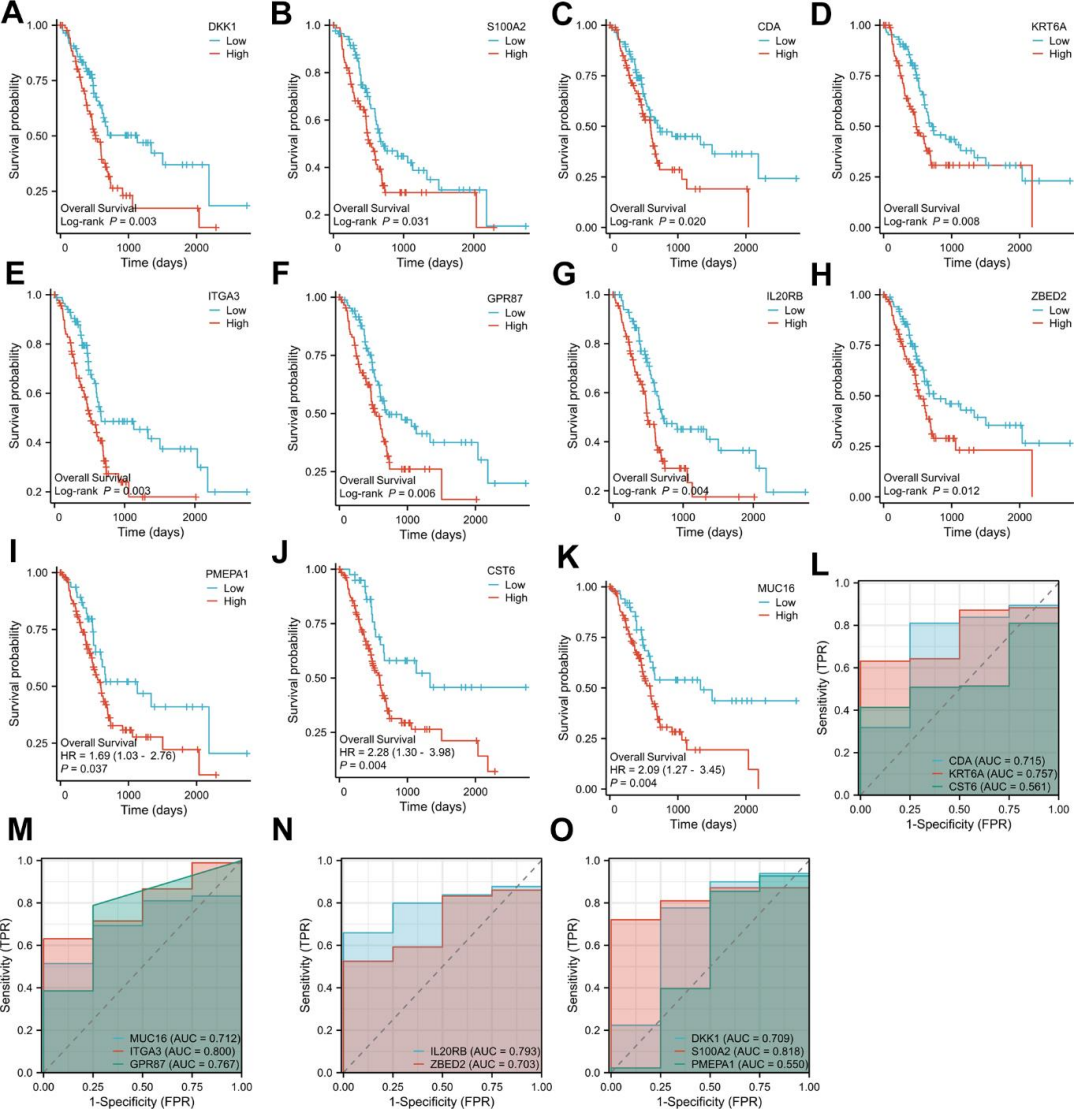
Overall survival analysis of the 11 key genes in PAAD (**A**–**K**); Diagnostic ROC plots based on the 11 key genes from pancreatic cancer data in the TCGA database (**L**–**O**).

We screened 11 key genes based on the principle of significant expression differences in GSE71989 (|log2FC|>1) and co-occurrence in the Navajowhite module, which were DKK1, S100A2, PMEPA1, CDA, KRT6A, CST6, MUC16, ITGA3, GPR87, IL20RB, and ZBED2. As illustrated in Figure 5A–5K, the levels of expression for 11 genes in PAAD tumor tissue were significantly higher in comparison to neighboring normal tissue. Based on the gene expression data from the TCGA database, diagnostic ROC curves and survival graphs were generated to assess the diagnostic usefulness of these genes for pancreatic cancer and survival. Survival graphs were constructed using the TCGA database to compare the prognosis of the higher and lower groups. The study findings suggest that the survival of pancreatic cancer patients is associated with the expression levels of 11 key genes (P-value < 0.05). (Figure 6A–6K). The AUCs were shown in Figure 6L–6O, which depicted the 11 key genes based on the ROC validation analysis on the validated TCGA dataset, namely, DKK1 (AUC=70. 9%), S100A2 (AUC=81. 8%), PMEPA1 (AUC=55%), CDA (AUC=71. 5%), KRT6A (AUC=75. 7%), CST6 (AUC=56. 1%), MUC16 (AUC=71. 2%), ITGA3 (AUC=80%), GPR87 (AUC=76. 7%), IL20RB (AUC=79. 3%) and ZBED2 (AUC=70. 3%).

### Related small molecule drug screening

We screened the CMAP database for natural small molecule drugs to identify potential drug candidates for pancreatic cancer. Natural small molecule drugs with high relevance based on the screened genes. The top 10 small molecule drugs were selected by norm_cs score (Table 1). Taxifolin, quercetin, and papaverine showed a high negative correlation among these small molecule drugs and had potential for treating pancreatic cancer. Some of these drugs have been shown to have anticancer effects. The top-ranked Taxifolin was selected as the target drug for further experimental validation.

**Table 1:** Results of CMAP analysis.

**RANK**	**Name**	**Moa**	**Norm_cs**
1	taxifolin	Opioid receptor antagonist	-1.9845
2	quercetin	Polar auxin transport inhibitor	-1.8074
3	papaverine	Phosphodiesterase inhibitor	-1.8072
4	fraxidin	Carbonic anhydrase inhibitor	-1.7963
5	caffeine	Adenosine receptor antagonist	-1.73
6	fumagillin	Methionine aminopeptidase inhibitor	-1.7221
7	arctigenin	MEK inhibitor	-1.7174
8	bilobalide	GABA receptor modulator	-1.7004
9	camptothecin	Topoisomerase inhibitor	-1.6964
10	piceatannol	Syk inhibitor	-1.675

### Inhibition of proliferation and clonogenicity of MIA PaCa-2 cells in pancreatic cancer by Taxifolin

**Figure 7:**
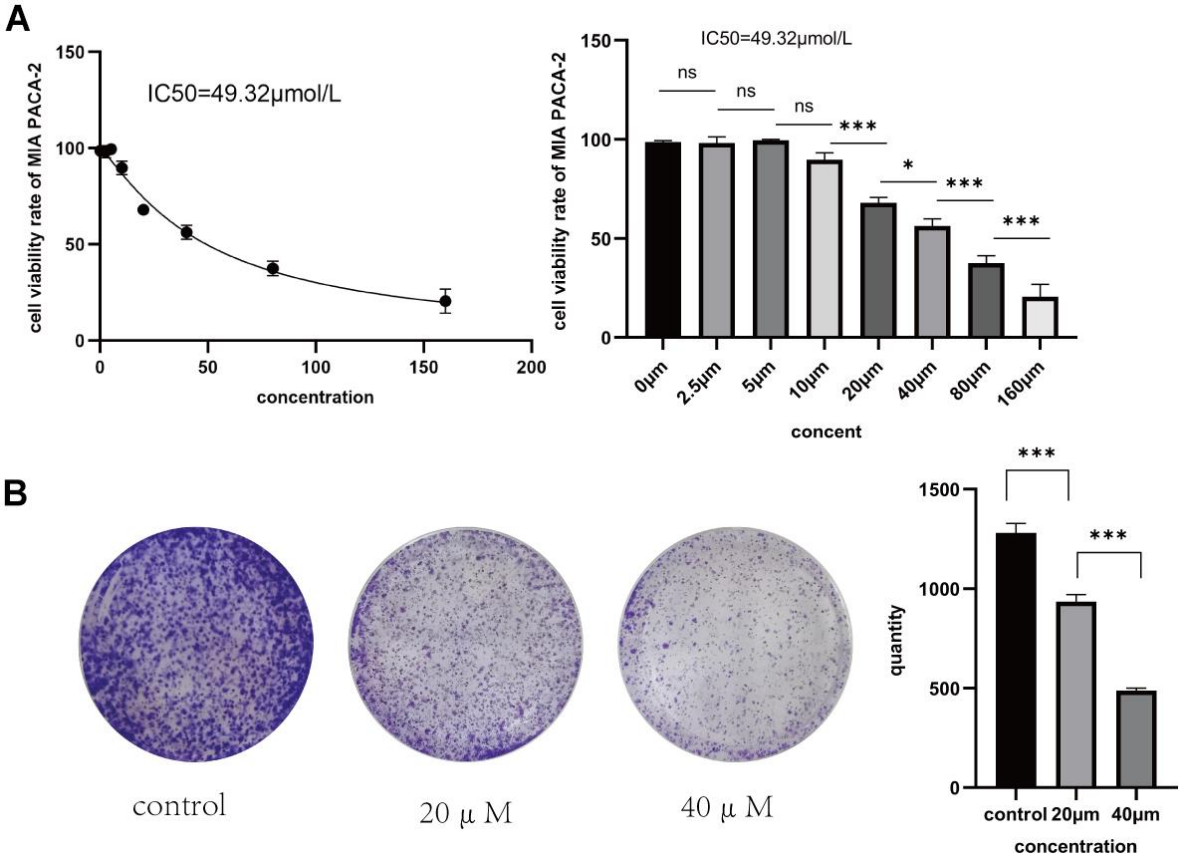
(**A**) Rate of proliferation inhibition in MIA PaCa-2 pancreatic cancer cells following 48 hours of Taxifolin treatment. (**B**) The impact of Taxifolin on the clonogenic potential of MIA PaCa-2 pancreatic cancer cells.

To confirm Taxifolin’s inhibitory effect on pancreatic cancer cells, MIA PaCa-2 cells were treated with Taxifolin at concentrations ranging from 0 to 160 μM. After 48 hours of incubation, a significant and dose-dependent inhibition of cell proliferation was observed in the MIA-PaCa-2 cells. The calculated half inhibitory concentration (IC50) of Taxifolin in MIA PaCa-2 cells at 48 h was 49. 32μM (Figure 7A). We conducted a clonogenic assay to examine Taxifolin’s clonogenic potential on pancreatic cancer cells. Results indicate that Taxifolin greatly inhibited the clone-forming ability of MIA PaCa-2 cells, with a gradual decrease in clone-forming ability as the drug concentration increased (see Figure 7B).

### Inhibition of migration and invasion of pancreatic cancer MIA PaCa-2 cells by Taxifolin

**Figure 8:**
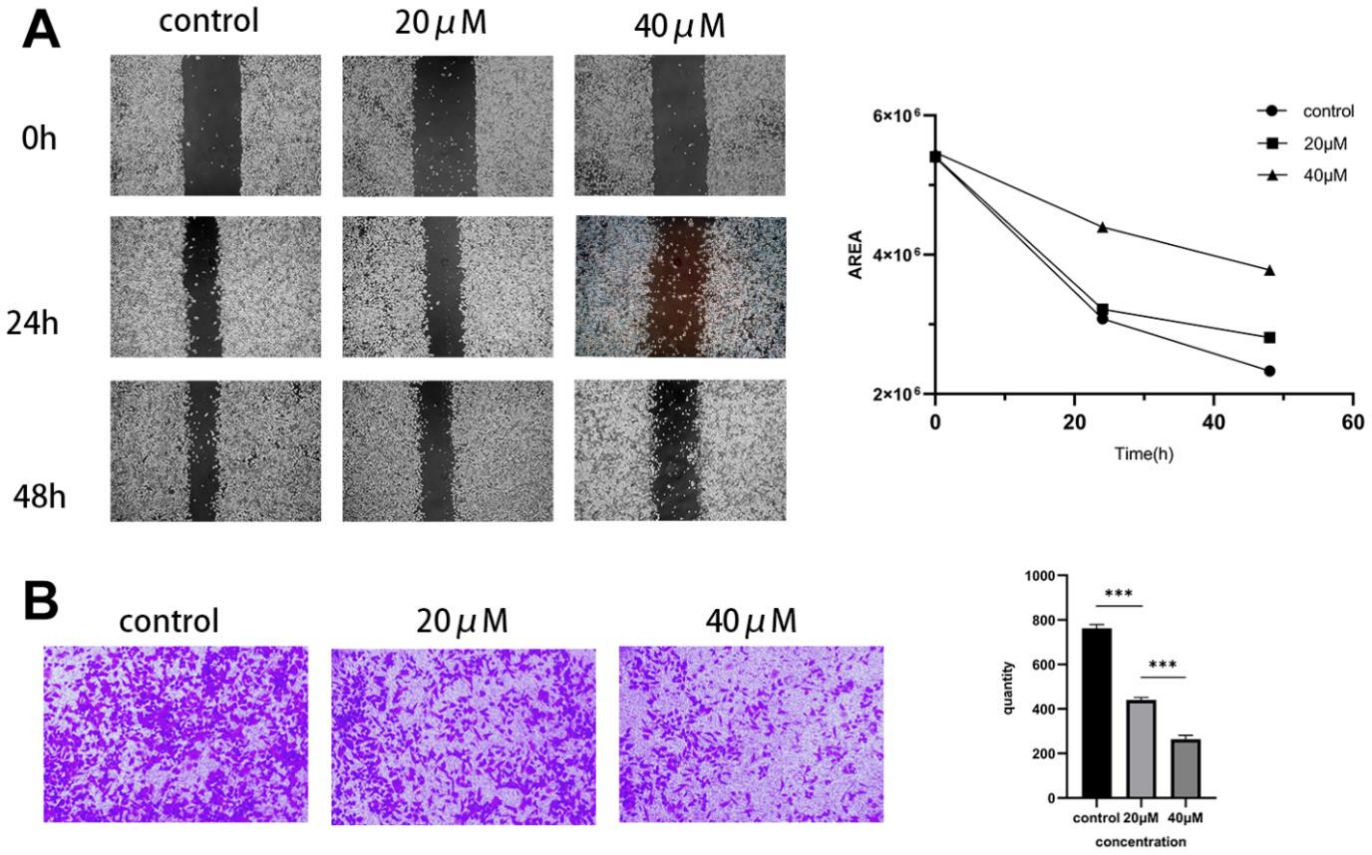
**Effect of Taxifolin on the immigration and invasion capacity of MIA PaCa-2 pancreatic cancer cells.** (**A**) Scratch assay to examine the migration of MIA PaCa-2 cells affected by Taxifolin. (**B**) Transwell assay was conducted to evaluate the effect of Taxifolin on the invasion of MIA PaCa-2 cells.

The previous experiments confirmed Taxifolin’s impact on the proliferative ability of pancreatic cancer cells MIA PaCa-2 through CCK-8 assay. In order to further evaluate Taxifolin’s effect on the migratory and invasive ability of these cells, we conducted scratch and Transwell assays. The study results showed that Taxifolin significantly inhibited the migration and invasion ability of MIA PaCa-2 cells. As the drug concentration increased, the cells’ ability to migrate decreased gradually (Figure 8A). Furthermore, a lower concentration of Taxifolin (20μM) significantly restricted cell invasion ability (Figure 8B).

### Taxifolin induced apoptosis of MIA PaCa-2 pancreatic cancer cells

**Figure 9:**
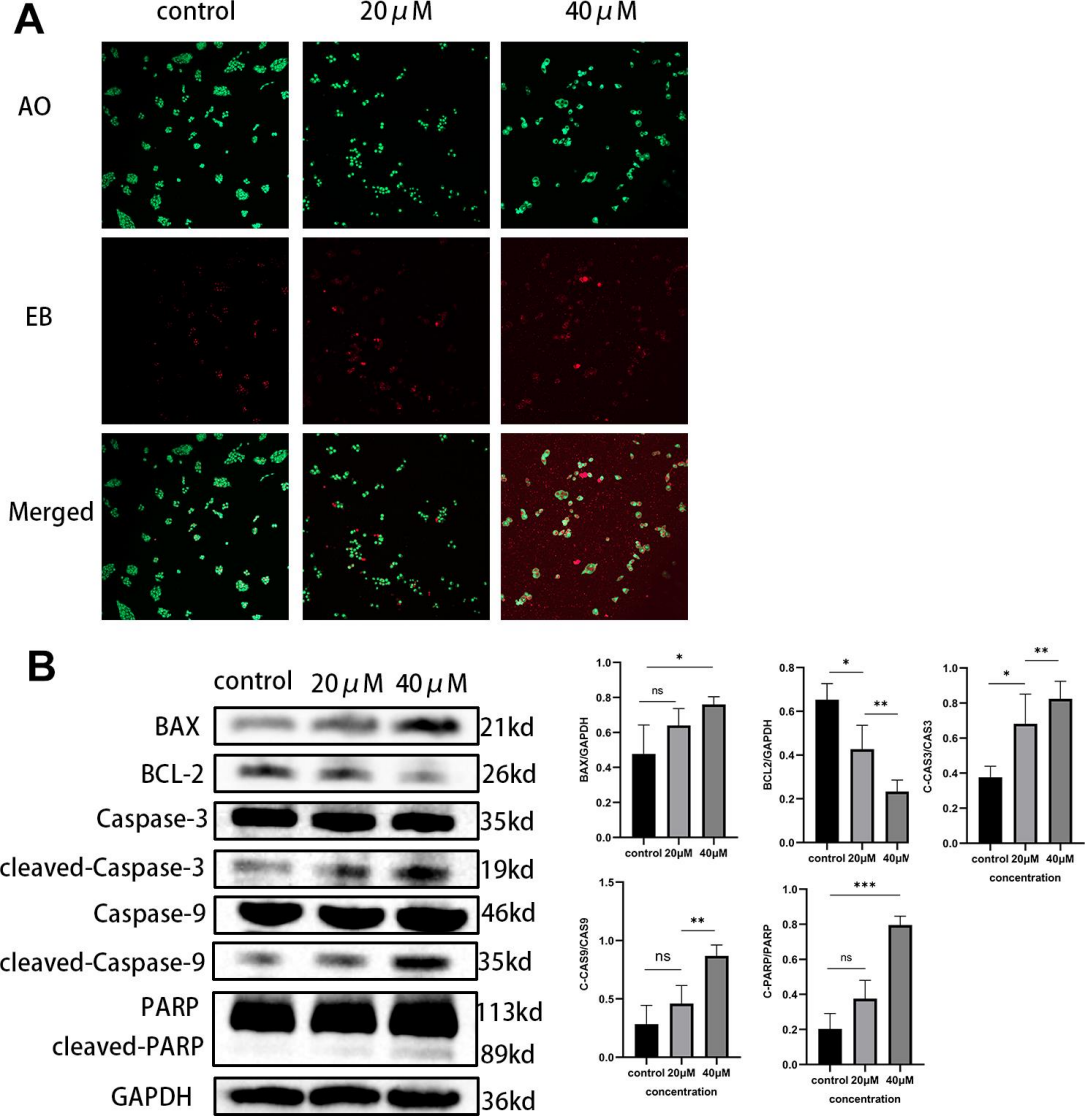
(**A**) MIA PaCa-2 cells were double-stained with AO/EB after Taxifolin treatment. (**B**) The expression of apoptosis-related proteins in MIA PaCa-2 cells after Taxifolin treatment.

Through the action of various drugs, the growth of tumor cells can be effectively inhibited and apoptosis was induced, thereby fulfilling the goal of treating cancer and improving the survival rate of patients. After treating MIA PaCa-2 cells with different concentrations of Taxifolin, we determined the proportion of apoptotic cells by AO/EB assay, which indicated that Taxifolin treatment resulted in obvious apoptotic changes (Figure 9A). Next, proteins related to apoptosis in pancreatic cancer cells after Taxifolin treatment were examined by Western blot. Statistical analysis of the gray-scale values of each band (Figure 9B) indicates that treatment with Taxifolin resulted in increased values of pro-apoptotic protein Bax and anti-apoptotic protein Bcl-2 in MIA-PaCa-2 cells (p<0.05). Meanwhile, Taxifolin treatment significantly increased the expression levels of PARP, caspase-9 and caspase-3. Analysis of the results showed that treatment with Taxifolin triggered both exogenous and endogenous apoptotic pathways.

### Screening information for Taxifolin targets

**Figure 10:**
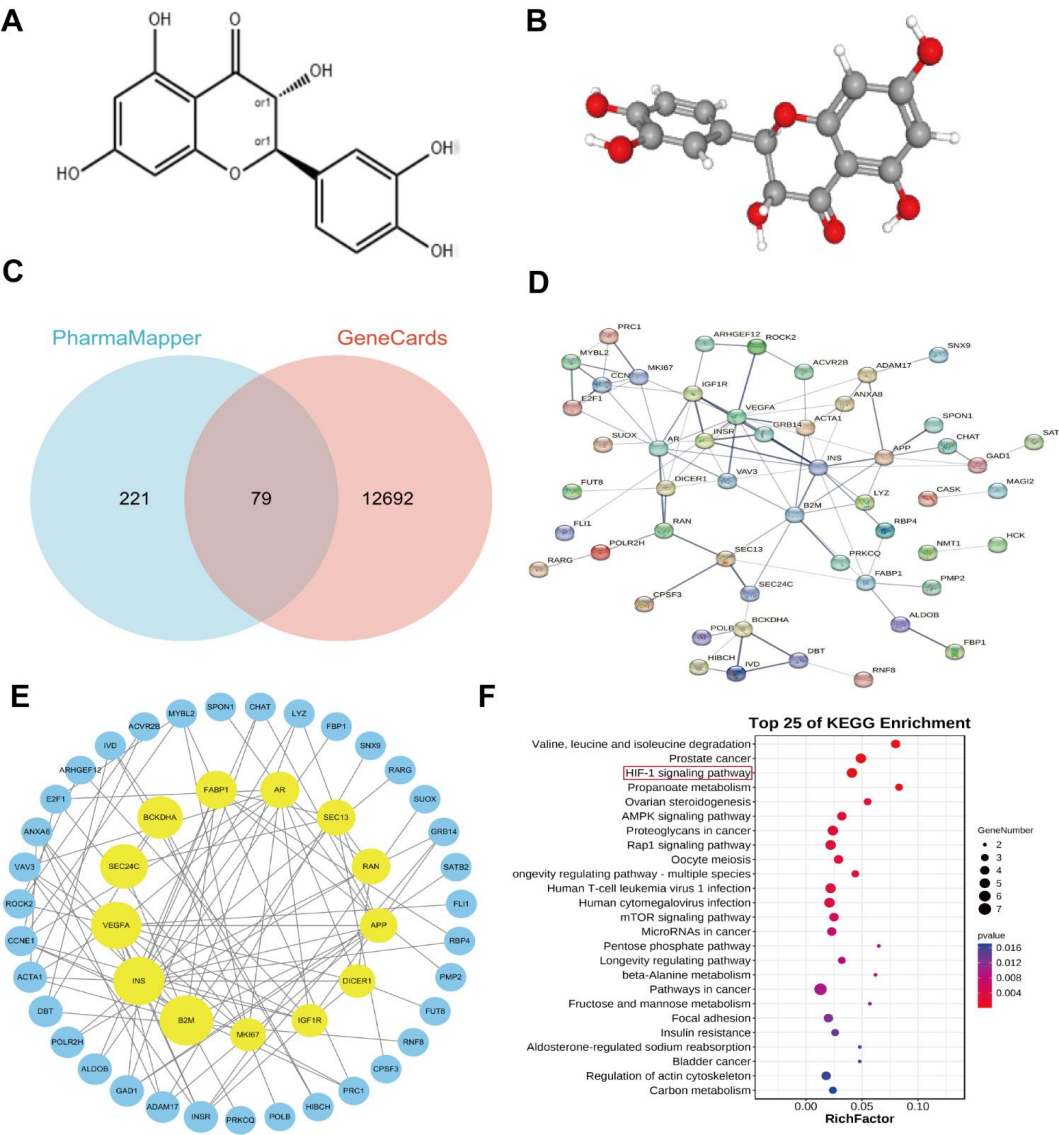
(**A**) Showed a 2D structural diagram of Taxifolin, (**B**) showed its 3D structural diagram. (**C**) Displayed the Interactive Wayne plots of the screening results obtained from the PharmMapper database and the GeneCards database. (**D**) Showed PPI interactions of the Taxifolin target genes. In (**E**) PPI plots of the Taxifolin target genes collated by the Cytoscape software were shown. (**F**) Displayed bubble plots of the KEGG pathway analyses of the key genes.

To better understand the potential apoptotic effects of Taxifolin on pancreatic cancer cells, we conducted a bioinformatics analysis. We retrieved the 2D and 3D chemical structures of Taxifolin from PubChem, as shown in Figure 10A, 10B, respectively. After uploading the structures to the PharmMapper database, we obtained a list of potential target proteins. From the top 300 target proteins, we selected 79 by intersecting them with a list of 12,771 genes related to pancreatic cancer from GeneCards. The final identified molecular targets are presented in Figure 10C.

### Protein interaction PPI network construction

To investigate the protein interaction network of Taxifolin’s potential target proteins, we utilized the STRING database platform to analyze the 79 proteins along with their annotated gene names. The results of the PP1 analysis (Figure 10D) showed that 52 of the nodes were interconnected, forming 95 edges, and 27 nodes were isolated. This suggested that the target did not interact with other proteins.

The PPI network arrangement revealed dense interactions among multiple targets within the core region. Calculation of the key topological parameters for the PPI network demonstrated that 52 nodes had an average degree value of 2. 5. Out of the 52 examined nodes, the genes B2M, INS, VEGFA, SEC24C, and BCKDHA had the most significant degree scores. This discovery indicates that these nodes may function as the key targets affecting the pharmacological impacts of Taxifolin. These 52 genes were organized using Cytoscape 3.7.1 software, and after removing the outlier genes, 48 key genes remained (Figure 10E).

### Analysis of KEGG signaling pathway of key genes

The 48 prime protein targets affected by Taxifolin were correlated with the list of KEGG-treated disorders and their associated primary signaling pathways. A comprehensive KEGG enrichment analysis evaluated a set of 37 genes involved in various signaling pathways, and found that they were associated with 136 diseases and signaling pathways.

The analysis identified several enriched pathways and diseases for the key target genes, including Valine, leucine, and isoleucine degradation, Prostate cancer, HIF-1 signaling pathway, Propanoate metabolism, and Ovarian steroidogenesis. Figure 10F highlighted the enrichment of the HIF-1 signaling pathway, propanoate metabolism, and ovarian steroidogenesis. Among these pathways, the HIF-1 signaling pathway was strongly associated with tumors. It was predicted that Taxifolin can promote apoptosis of pancreatic cancer cells through HIF-1 signaling pathway.

### Predicted binding patterns of Taxifolin to important target proteins

**Figure 11:**
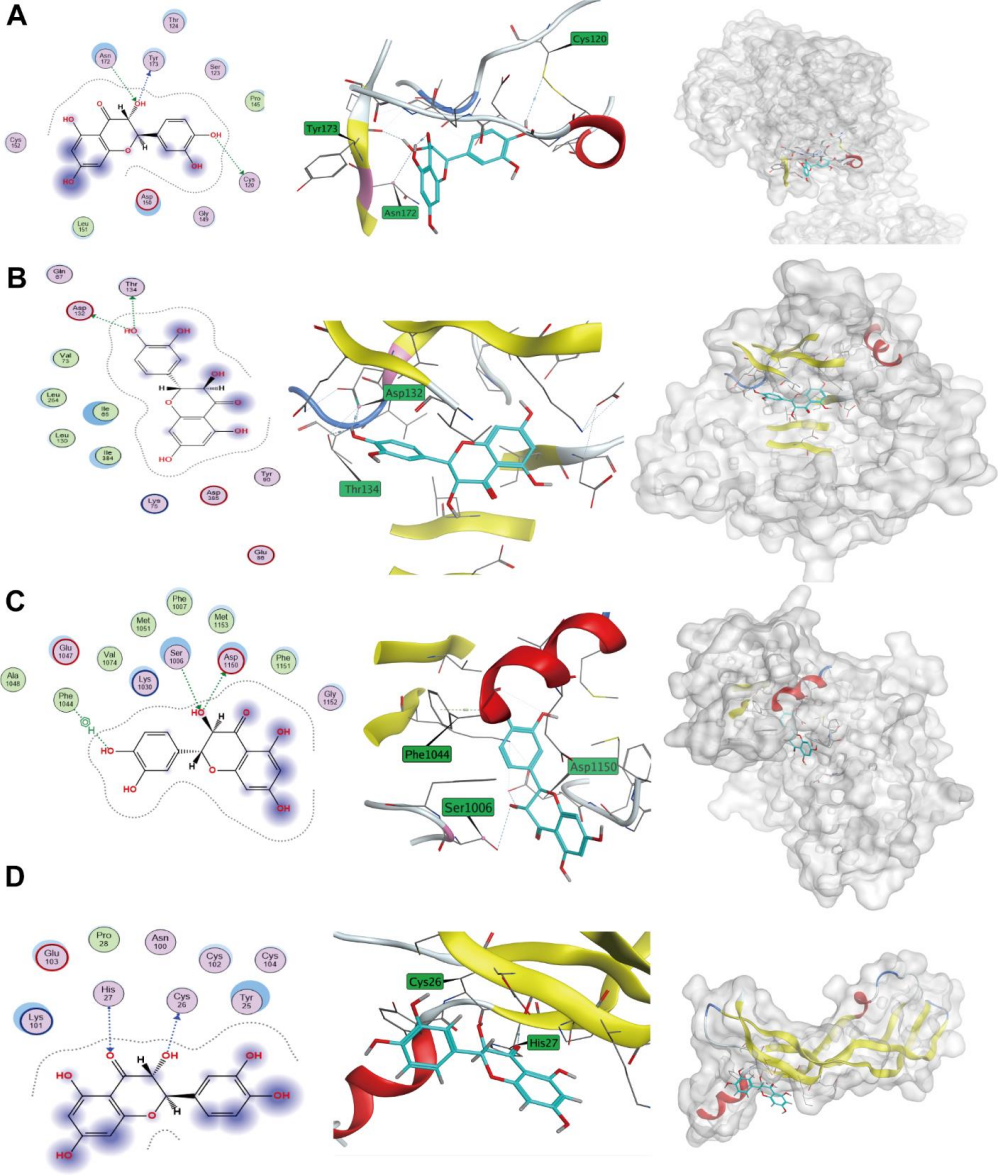
**Taxifolin-target protein binding patterns.** (**A**) IGF1R; (**B**) INS; (**C**) INSR; (**D**) VEGFA.

KEGG enrichment analysis identified crucial nodes in the signaling pathways associated with cancer development, including VEGFA, INS, IGF1R, and INSR. Furthermore, we retrieved the respective structure files of the identified targets from the PDB database. The MOE software was utilized to carry out forward molecular docking between Taxifolin and the target. The results were presented in Figure 11. Using MOE molecular docking software, we calculated the binding-free energy of each target, and observed that four protein targets exhibited binding potential with Taxifolin.

Among the four targets, the amino acid sites where Taxifolin mainly acted on IGF1R protein included Tyr173, Asn 172, Cys120 (Figure 11A). The amino acid sites where Taxifolin mainly acted on INS protein included Asp132, Thr134 (Figure 11B). The amino acid sites where Taxifolin mainly acted on INSR protein included Phe1044, Ser1006, Asp1150 (Figure 11C). The amino acid sites on VEGFA protein where Taxifolin mainly acted included His27, Cys26 (Figure 11D). The MOE software was utilized to compute the binding free energy of every individual target binding site, and the results showed that all the four protein targets had binding potential with Taxifolin (Table 2).

**Table 2:** The results of Taxifolin-target protein docking.

**Target points**	**PDB ID**	**Combined free energy (Kcal/mol)**
VEGFA	4KZN	-4.9668
INS	5W2H	-5.5051
IGF1R	8EYR	-5.0767
INSR	5E1S	-5.5836

## DISCUSSION

Cancer is currently a significant global public health problem [15–17]. Patients with pancreatic cancer typically survive for about 10-12 months after treatment due to late diagnosis and a restricted pool of patients who qualify for surgical resection [18, 19]. The standard treatment for pancreatic cancer includes FOLFIRONOX (a blend of 5-fluorouracil, calcium folinic acid, irinotecan, and oxaliplatin) or gemcitabine with albumin-conjugated paclitaxel [20, 21]. Nevertheless, metastasis occurs early in the progression of pancreatic cancer and is prone to chemotherapeutic drug resistance, thereby posing a significant challenge to clinical treatment [22]. Hence, further exploration of novel prognostic biomarkers, therapeutic targets, and therapeutic agents is crucial to enhance the therapeutic approach for pancreatic cancer.

WGCNA is a technique employed for characterizing gene associations across samples. It detects gene sets with high synergy, which can then be sorted into clusters based on comparable gene expression patterns. Subsequently, these clusters can be analyzed further to comprehend the connections among them and specific features, such as the patients’ clinical information. Additionally, WGCNA can screen potential biomarkers or therapeutic targets by analyzing interactions between gene sets and associations of gene-set with phenotypes [23–25]. Yongwen et al. [26] applied WGCNA to identify six modules related to clear cell renal cell cancer (ccRCC) pathology staging and grading, and identifies a total of nine key genes related to ccRCC patient progression and prognosis. Multivariate Cox regression analysis revealed that risk scores founded on the attributes of the nine pivotal genes were clinically autonomous prognosticators for ccRCC patients. Zohreh et al. [27] employed WGCNA to screen biomarkers for early detection of stomach cancer and found that ITGAX, CCL14, ADHFE1, HOXB13 were higher in stomach cancer tumor tissues than in normal neighboring tissues. Further approaches in systems biology have also confirmed the potential of such genes as gastric cancer marker genes. Hence, WGCNA is a trustworthy technique for detecting the disease’s key targets.

In this study, we identified 11 significant genes linked to patient outcome and prognosis using WGCNA analysis. Their diagnostic efficiency in pancreatic cancer was verified, and their correlation with patient prognosis was evaluated using OS and diagnostic ROC analyses based on the GEPIA and TCGA databases. Some of the 11 genes were proven to be highly significant in pancreatic cancer. Integrin Subunit Alpha 3 (ITGA3) gene has a close association with pancreatic cancer [28]. ITGA3 expression was observed to be higher in pancreatic cancer patients compared to non-cancer patients and showed correlation with clinical traits such as histologic type, histologic grade, stage, T classification, and survival status. Higher expression of ITGA3 in pancreatic cancer patients, particularly in early-stage cancer patients, is associated with poorer overall survival and recurrence-free survival [29, 30]. Hence, ITGA3 has the potential to serve as a diagnostic marker and prognostic indicator for pancreatic cancer. DKK1 is another marker associated with a wide range of tumors, and the dysregulated expression of this gene has been linked to the development and progression of several cancers, including breast cancer [31, 32]. DKK1 is considered a promising biomarker for the early diagnosis and prediction of pancreatic cancer and can be used by WGCNA to screen for key pancreatic cancer targets.

The Connectivity Map (CMAP) archives differences in gene expression of human cells after exposure to different perturbations and their corresponding biological applications. To create the database, the drug sequencers first record the gene expression profiles of thousands of drugs after treating various cells [33]. Then we can upload our list of differentially expressed genes to compare it with the database reference dataset using CMAP and screen small molecule drugs that may show efficacy based on gene expression differences. A previous study used WGCNA to filter genes associated with endometriosis and macrophages, and the study obtained 23 small molecules from the CMAP database with potential therapeutic effects on endometriosis [34]. In another study, the DGIdb/CMAP database was utilized to anticipate appropriate drugs for Alzheimer’s disease and rosacea. After prediction, Melatonin (MLT) was chosen as the targeted drug [35]. Melatonin’s (MLT) therapeutic effects and mechanisms in rosacea were verified *in vivo* and *in vitro*, providing a solid theoretical basis for our study.

Small molecule drugs derived from natural products offer unique advantages in the treatment of tumors [36]. One of the advantages of natural products are their diversity, which allows us to discover a range of compounds with anti-tumor activity, providing wider options for tumor treatment. Moreover, small molecule drugs from natural products have high efficiency and specificity, enabling selective targeting of tumor cells and a lower risk of damage to healthy cells. Furthermore, in comparison to large molecule drugs, small molecule drugs derived from natural products typically have less severe side effects and suit better for long-term use. However, certain small molecule drugs present in natural products have demonstrated resistance against multidrug-resistant tumor cells, thus showing potential in treating refractory tumors. Also, natural products are comparatively easy to be obtained, presenting a promising source of medication in tumor therapy. To summarize, the benefits of small molecule drugs extracted from natural products in tumor therapy have high efficiency, specificity, accessibility as well as less side effects and drug resistance, which allow them to be exploited as promising drug candidates in tumor therapy.

The top-scoring natural small molecule drug Taxifolin was screened using the CMAP combined with norm-cs scoring. Taxifolin is a flavonoid found in various plants and exhibits a diverse range of biological actions. Taxifolin has been widely employed in various fields such as food, medicine, and industry. Taxifolin could boost the human immune system and contribute to disease prevention. Additionally, it has antioxidant, anti-inflammatory, and anti-tumor effects [37]. Ronghua Wang et al. [38] found that Taxifolin could inhibit the stemness and EMT of lung cancer cells by inhibiting the inactivation of PI3K and OCT4, demonstrating its potential of a viable therapeutic agent for lung cancer. Chen Xin et al. [39] reported that Taxifolin, derived from yew, could inhibit the growth, migration, and invasion of human osteosarcoma cells. Nevertheless, the effectiveness and mechanism of Taxifolin in treating pancreatic cancer remain unclear.

Through implementation of CCK8, clonogenic assays, and Transwell experiments, it has been successfully validated that Taxifolin is capable of suppressing the proliferation, migration, and invasion of pancreatic cancer cells. Several previous studies have found that Taxifolin enhances mesenchymal-epithelial transition via the β-catenin signaling pathway. At the same time, it inhibits the ability of breast cancer cells to multiply, invade, and migrate, which was in line with our findings [40]. Furthermore, various scholars have shown that Taxifolin can induce apoptosis of cancer cells, including cervical and prostate cancer cells. Our study found that Taxifolin induced apoptosis of pancreatic cancer cells, as confirmed by the AO/EB assay and changes in expression levels of apoptosis-related proteins detected by Western blot. In a subsequent study, we predicted that inhibiting pancreatic cancer progression using Taxifolin was achieved through the HIF-1 signaling pathway based on network pharmacology analysis. This was consistent with the results of Butt et al. [41]. The pro-apoptotic activity of Taxifolin was confirmed in the hepatocellular carcinoma cell lines HepG2 and Huh7. It was shown to induce apoptosis. The down-regulation of HIF-1 expression in a dose-related manner indicates that taxifolin exerts its anti-tumor effect through the HIF-1 signaling pathway as one of its mechanisms. However, the signaling pathway was not thoroughly investigated in pancreatic cancer, which became a limitation of the current study. Further studies including *in vivo* investigations on the anticancer effects of Taxifolin are required, specifically in pancreatic cancer. We hope future studies could further improve the findings of our study.

In summary, the combination of WGCNA and CMAP was a reliable method in discovering disease-related key genes and small molecule drugs that may have therapeutic activity. On the basis of this method, Taxifolin has been identified as a possible drug for the treatment of pancreatic cancer.

## MATERIALS AND METHODS

### Gene expression profiling data

We obtained mRNA gene expression profiles and corresponding clinical data for pancreatic cancer from the Gene Expression Omnibus (GEO) database (http://www.ncbi.nlm.nih.gov/geo/). The dataset GSE71989 includes 13 samples of pancreatic cancer tissues and 9 normal pancreatic tissues. Gene expression profiling was used to identify differentially expressed genes. We obtained data related to pancreatic cancer, including gene sequencing data and corresponding clinical information, from The Cancer Genome Atlas (TCGA) database, using the University of California, Santa Cruz (UCSC) Genome Browser. We used this data to perform WGCNA and validate core genes.

### Data pre-processing and differential expression gene screening

The data set GSE71989 was retrieved from the GEO database using the GEOquery package of the software “R” (4.2.1) and normalized using the normalizeBetweenArrays function of the limma package. The samples were viewed in box plots, the clustering between the sample groups was viewed in PCA plots, and the two data sets were analyzed for differences using the Limma package. The results of the difference analysis were visualized using volcano plots.

### WGCNA

We constructed a weighted gene co-expression network using the R software package “WGCNA” and performed sample clustering. Subsequently, we determined the soft-threshold power β by using a standard scale-free network. Then, using the power adjacency function of the Pearson correlation matrix, we calculated the connectivity between all filtered genes. We converted the data to a topological overlap matrix (TOM) and calculated the corresponding dissimilarity (1-TOM). A gene dendrogram was generated based on the dissimilarity measure using TOM. Clustering was performed on the average linkage hierarchical structure with a minimum value of 50. We computed the gene differences between the representative modules and identified and merged highly similar modules based on clustering.

### Identification and functional annotation of clinically important modules

Module eigengenes (ME) are defined as the major components of a given module, representing the gene expression profile within the module. We calculated the correlation between ME and clinically significant features to identify relevant modules and provide a summary. Gene significance (GS) is defined as the logarithm to the base 10 (log10) transformation of the P-value. It is obtained from a linear regression analysis conducted between gene expression and pathological progression. The module significance (MS) is calculated as the average gene significance (GS) within a module. The module with the highest absolute value of MS is considered to be the one that correlates with clinically significant traits among the selected modules. To explore the potential mechanisms of how the module genes affected relevant clinical traits, we uploaded all genes in Navajowhite modules to the Database Annotation, Visualization, and Integrated Discovery (DAVID) online tool (https://david.ncifcrf.gov/). We performed a functional enrichment analysis using the Gene Ontology (GO) pathway database and the Kyoto Encyclopedia of Genes and Genomes (KEGG) pathway database, with a significance threshold of P < 0.05.

### Identification and validation of hub genes

We extracted the genes from the Navajowhite module. Then, we identified significant gene expressions (|log2FC|>1) in GSE71989 that also co-occurred in the major modules. Further, the reliability and accuracy of the hub genes were confirmed by ROC curves using the TCGA database. Survival curves were established based on the data from the GEPIA database.

### Screening of small molecule drugs using the CMAP database

CMAP, also known as Connective Map, is a database for gene expression profiling. After uploading our hub gene data for pancreatic cancer, we compared it with the reference dataset available on CMAP to search for small molecule drugs based on expression comparison. Therapeutic drugs for pancreatic cancer were selected using norm-cs scoring sequences.

### Taxifolin information

Taxifolin, registered under the Chemical Abstracts Service (CAS) number 480-18-2, possesses a molecular formula of C15H12O7 and a molecular weight of 304.25 g/mol. Its structural data were obtained from the PubChem organic small molecule bioactivity database (https://pubchem.ncbi.nlm.nih.gov/). The standard Taxifolin was acquired from MedChemExpress (Monmouth Junction, NJ, USA) and stored at room temperature. The stock solution of Taxifolin was prepared by reconstituting the mother liquor to a concentration of 20mM using dimethyl sulfoxide (DMSO), and it was subsequently stored at -20° C for future use.

### Cell culture

The MIA PaCa-2 pancreatic cancer cell line is of human origin. It was acquired from Sevier Biologics (Wuhan, China). The cells were cultured in high-glucose DMEM medium supplemented with 1% penicillin-streptomycin (Thermo Fisher Scientific, Waltham, MA, USA) and 10% FBS (VivaCell Bioscience, Shanghai, China). The cells were cultured in a CO2 incubator at 37° C with 95% air and 5% CO2, and the culture medium was replaced two to three times per week.

### CCK-8 experiment

The human pancreatic cancer cell line MIA-PaCa-2 was cultured to logarithmic growth phase, and a single-cell suspension was prepared using trypsin (Solarbio Life Science, Beijing, China). A counting plate was used to regulate the cell density, and 5 replicate wells per group with 5000/100 μl plates were set up in 96-well plates. To reduce the effects of evaporation, PBS buffer was included in the edge wells prior to overnight incubation. Taxifolin was diluted to the target concentrations (2. 5, 5, 10, 20, 40, 80, 160 μmol/L) the following day. The DMSO concentration in the 160 μmol/L Taxifolin was utilized as a negative control. The liquid in the plate was removed. DMSO and Taxifolin were then given to each of the wells at the above concentrations. A control group was included as a reference. The plates were subsequently incubated for 48 hours (h). Next, 10 μL of CCK-8 solution (Toho Chemical, Japan) was given to each hole in the plates, which were returned to the incubator for one hour. After incubation, we measured the Optical Density (OD) at 450 nm using an enzyme-labeled instrument (Molecular Devices, Shanghai, China). The obtained results were subsequently analyzed and processed.

### Clone formation experiments

Cells in logarithmic growth phase were harvested from the MIA PaCa-2 cell line, and single cell suspension were prepared for cell counting. The cells were diluted using complete medium and plated in 6-well plate at a density of 1500 cells in each well. Once the cells attached to the plate surface, the reference group received complete medium containing DMSO, while the remaining cells were treated with either 20 or 40 μmol/L Taxifolin for 48h. The complete culture medium was changed and replenished every 2 days for a duration of 14 days following treatment. Once the cells formed clusters, they were fixed by 4% paraformaldehyde (Leagene Biotechnology, Beijing, China) and stained by 0. 1% crystal violet (Solarbio Life Science, Beijing, China) to count the number of clones.

### Scratch experiment

A suspension of MIA-PaCa-2 cells was prepared and distributed it onto a 6-well plate. The following day, once the cells had adhered to the wall, a line perpendicular to the mark on the back was drawn in the wells using a 200-μL Pipette gun head. Subsequently, the floating cells were rinsed off using PBS. The control group was given a medium of 2% FBS for incubation, and the experimental group was given 2% FBS with the medium of Taxifolin diluted to 20 and 40μM. The cells were photographed under a microscope, incubated, and photographed again at the 24 and 48-hour marks. The size of the scratches was measured with Image J software version 2.1.0 (Bethesda Softworks, Bethesda, MA, USA).

### Transwell experiment

Before the Transwell experiment, the cells were cultured in a 2% FBS medium for 12 hours. Matrix gels (BD Biosciences, New York, NK, USA) were placed on ice to dissolve, and a precooled lance was used to add 50 μL of matrix gel to each upper chamber of the Transwell (Corning Life Science, New York, NY, USA) in the matrix gel. The chambers were incubated at 37° C overnight to allow full solidification in the matrix gel. On the second day, 800μL of complete culture medium containing 15% FBS was added to the lower chamber of the Transwell. Cells were diluted to 4 × 10^4 cells/200μL in culture medium with 2% FBS or varying concentrations of Taxifolin. 200μL of the prepared cell suspension was added to the upper chamber of the Transwell placed in the incubator. After 48 hours, we fixed the Transwell chamber with 4% paraformaldehyde at 15 minutes, followed by staining with 0.1% crystal violet solution at 15 minutes. Next, wash the compartment twice with PBS. Then gently remove the matrix gel and cells in the upper chamber with a cotton swab. Finally, the chamber was placed under a microscope to capture pictures of 4-5 random fields of view. The average cell count in each visual field was then computed.

### Acridine orange-ethidium bromide (AO/EB) staining to detect apoptosis

MIA-PaCa-2 cells were prepared by creating a single-cell suspension and counting them. The cell concentration was adjusted to 1×10^5/ml and inoculated them into a confocal dish. Following cell attachment, the cells were subjected to treatment with DMSO and varying concentrations of yew Taxifolin for a duration of 48 hours. The supernatant was discarded after adding 2μL AO/EB working solution (Faraway Wildlife, Shanghai, China). The cells were blended and allowed to incubate for 15 minutes at room temperature, away from light, and quickly photographed under a fluorescence microscope.

### Western blot experiment

Cells were seeded in diameter Petri dishes (10 cm) and treated with DMSO and Taxifolin, respectively. After treatment completion, the cells were lysed with lysis solution (Solarbio Life Science, Beijing, China) which contained 1% PMSF (by volume) and a phosphatase inhibitor. The lysate was then disrupted by ultrasound and then subjected to centrifugation at 12,000 rpm for 10 minutes in 4° C. After centrifugation, the supernatant should be extracted and the concentration should be detected using the BCA quantitative reagent kit (Solarbio Life Science, Beijing, China). An appropriate volume of 5x loading buffer was then dispensed and the mix was boiled for 10 minutes, followed by storage at -20° C in a refrigerator. For protein content detection, SDS-PAGE gels were prepared at 10% or 7.5% concentration depending on the molecular weight size, and the samples were added for electrophoresis and then transferred to the PVDF membrane, followed by incubation first with 5% nonfat dry milk at ambient temperature for 1.5 h, then with the corresponding primary antibody on a shaker at 4° C overnight, and with the secondary antibody at ambient temperature the next day. Antibody was developed using ECL exposure solution. Image J software 2. 1. 0 was used for grey value calculation.

The following antibodies were obtained from Proteintech Group (Wuhan, China) for use in this study:

GAPDH (Proteintech, #10494-1-AP, 1:5000); HRP-conjugated affinipure rabbit anti-goat IgG (Proteintech, #SA00001-2, 1:6000); Bcl-2 (Proteintech, #60178-1-lg, 1:4000); PARP & cleaved-PARP (Proteintech, #66520-1-lg, 1:10000); HRP-labeled Goat anti-mouse IgG (Proteintech, #PR30012, 1:6000), Caspase3 (Proteintech, #66470-1-Ig, 1:3000); Caspase9 (Proteintech, #66169-1-Ig, 1:2000); Bax (Proteintech, #60267-1-Ig, 1:20000).

### Screening of potential targets of Taxifolin

The three-dimensional structural pattern map of Taxifolin was submitted to PharmMapper (http://www.lilab-ecust.cn/pharmmaDper/), an online website for structural target analysis and screening of pharmacophore. We selected a library of pharmacophore models for screening. To identify the relevant genes associated with pancreatic cancer, we downloaded the gene data obtained from the GeneCards database (https://www.genecards.org/). The intersection of two results yields the target gene.

### Protein interaction PPI network

We entered the predicted target proteins of Taxifolin from PharmMapper and GeneCards, along with the corresponding gene names, into the protein-protein interaction (PPI) online analysis database STING to examine the network of protein interactions of each drug target. The findings were then imported into Cytoscape 3.9 software for further analysis.

### Annotated analysis of key target functions and signaling pathways

The key protein targets of Taxifolin obtained from PPI analysis were uploaded and mapped to the corresponding disease catalogues and key pathways in the KEGG to construct a “component-target-pathway-disease” network. The target proteins showed different levels of enrichment in different pathways, and the enrichment level of KEGG was measured by Rich factor, P-value analysis in this study.

### Prediction of positive molecular docking of Taxifolin with key target proteins

We employed KEGG enrichment analysis to identify pathways associated with cancer development. The target protein nodes in the target pathways were identified, and the corresponding target structure maps were downloaded from the PDB database. Dimerized proteins were subjected to structural preprocessing to obtain the 3D structures of the targeted proteins. The Molecular Operating Environment (MOE, Chemical Computing Group Inc, Quebec, Canada) docking software was used to conduct forward molecular docking between Taxifolin and the targets. The binding-free energy value of the receptor and ligand after binding was calculated, with a lower value indicating a stronger affinity between receptor and ligand.
